# Deciphering the Osteoimmune Landscape in Subtalar Arthrodesis: A Single‐Cell RNA Sequencing Approach

**DOI:** 10.1111/jcmm.70980

**Published:** 2025-12-11

**Authors:** Hansong Lee, Kihun Kim, Tae Sik Goh, Suk‐Woong Kang, Jung Yun Bae, Su‐Yeon Cho, Yujin Kwon, Won Kyu Kim, Jin‐Woo Kim, Yun Hak Kim, Seung Hun Woo

**Affiliations:** ^1^ Medical Research Institute Pusan National University Yangsan Republic of Korea; ^2^ Department of Occupational and Environmental Medicine Pusan National University Yangsan Hospital Yangsan Republic of Korea; ^3^ Research Institute for Convergence of Biomedical Science and Technology Pusan National University Yangsan Hospital Yangsan Republic of Korea; ^4^ Department of Orthopaedic Surgery Pusan National University Busan Republic of Korea; ^5^ Department of Orthopedic Surgery, Research Institute for Convergence of Biomedical Science and Technology, Pusan National University Yangsan Hospital Pusan National University School of Medicine Yangsan Republic of Korea; ^6^ Division of Natural Product Applied Science University of Science and Technology (UST) Daejeon Republic of Korea; ^7^ Center for Natural Product Efficacy Optimization Korea Institute of Science and Technology (KIST) Gangneung Republic of Korea; ^8^ Department of Convergence Medicine Yonsei University Wonju College of Medicine Wonju Republic of Korea; ^9^ Department of Oral and Maxillofacial Surgery, Research Institute for Intractable Osteonecrosis of the Jaw Ewha Womans University College of Medicine Seoul Republic of Korea; ^10^ Department of Anatomy, School of Medicine Pusan National University Yangsan Republic of Korea; ^11^ Department of Biomedical Informatics, School of Medicine Pusan National University Yangsan Republic of Korea

**Keywords:** monocyte, NK cell, osteoimmunology, single‐cell RNA sequencing, subtalar arthrodesis

## Abstract

Subtalar arthrodesis (SA) is a widely used salvage procedure for posttraumatic subtalar arthritis (PSA), but its underlying healing mechanisms remain poorly understood. The immune system plays a critical role in the union process after arthrodesis; however, the systemic osteoimmunological response has not been clearly defined. In this study, we investigated immune cell dynamics in patients who underwent SA using single‐cell RNA sequencing (scRNA‐seq). Peripheral blood mononuclear cells (PBMCs) were collected before surgery and 3 months after the operation. The degree of bone fusion was assessed using computed tomography (CT) scans at 3 months, and patients were categorised into early union (EU) and delayed union (DU) groups. scRNA‐seq analysis was performed to examine immune cell composition, gene expression and functional pathways. Monocytes in the EU group showed enhanced antigen processing and presentation, whereas those in the DU group demonstrated increased phagocytic activity. NK cells in the DU group exhibited stronger cytotoxicity through Fc‐gamma receptor signalling and antibody‐dependent cellular cytotoxicity (ADCC) pathways, while NK cells in the EU group showed higher chemokine and cytokine activity. These immune differences persisted postoperatively, suggesting that variations in the systemic immune environment may influence bone healing outcomes. Our findings emphasise the important roles of monocytes and NK cells in bone union and suggest that immunomodulatory approaches could help improve bone repair.

**Abbreviations**
ADCC: Antibody‐Dependent Cellular Cytotoxicity
AO: Acridine Orange
BSA: Bovine Serum Albumin
ClassicM: Classical Monocytes
CT: Computed Tomography
DEG: Differentially expressed gene
DIACF: Displaced intra‐articular calcaneus fracture
DMEM: Dulbecco's Modified Eagle Medium
DU: Delayed Union
EU: Early Union
FBS: Fetal Bovine Serum
FCGR3A: Fc Gamma Receptor IIIa
GO: Gene Ontology
IntermediateM: Intermediate Monocyte
PACS: Picture Archiving and Communication System
PBMCs: Peripheral Blood Mononuclear Cells
PBS: Phosphate‐Buffered Saline
PC: Principal Component
PCA: Principal Component Analysis
PI: Propidium Iodide
PSA: Posttraumatic Subtalar Arthritis
SA: Subtalar Arthrodesis
ScRNA‐Seq: Single‐Cell RNA Sequencing
SNN: Shared Nearest Neighbour
TNF: Tumour Necrosis Factor
UMAP: Uniform Manifold Approximation and Projection
UMI: Unique Molecular Identifier

## 1 Introduction

Subtalar arthrodesis (SA) is a commonly performed surgical treatment for posttraumatic subtalar arthritis (PSA) a condition that frequently develops following displaced intra‐articular calcaneus fracture (DIACF) [1]. Calcaneus fracture is the most common fracture of the tarsal bones, accounting for approximately 2% of all fractures [2]. Among these, DIACF involving the subtalar joint is 56%–75%, most of which require surgical intervention [3]. Patients managed conservatively have a sixfold higher risk of subsequently requiring SA [4]. Nevertheless, even after surgical management, PSA often persists and may require additional procedures [5].

The primary objective of SA is pain relief, which is achieved through the successful bony union of the subtalar joint [1]. Bony union stabilises the arthrodesis site, preventing shear forces and reducing intra‐articular peak forces, ultimately improving daily function [1]. Despite its widespread use, SA has high nonunion rates, ranging from 5%–45%, depending on subgroup analysis, bone grafting techniques and diagnostic modalities [6, 7, 8, 9, 10, 11, 12, 13]. Nonunion can lead to chronic pain, mobility impairment, and decreased quality of life, often requiring revision surgery, prolonged immobilisation and work disability [14]. Therefore, understanding the factors contributing to nonunion and developing strategies to enhance bone healing are of critical importance.

Bone healing is a highly coordinated biological process regulated by immune cells, signalling molecules and skeletal cells. Transcriptomic approaches, particularly Single‐Cell RNA Sequencing (scRNA‐seq), have provided valuable insights into the immune landscape by identifying distinct cell subtypes, quantifying cell‐type frequencies and identifying gene expression networks involved in bone remodelling [15]. While previous studies have explored osteoimmunology in fracture healing, no research has specifically investigated systemic immune responses in SA patients using scRNA‐seq [16, 17, 18, 19]. Additionally, many prior studies have been limited by small sample sizes and a lack of pre‐ and postoperative comparative analyses.

Therefore, this study aimed to elucidate the systemic osteoimmune landscape in SA patients by analysing peripheral blood mononuclear cells (PBMCs) before and after surgery using scRNA‐seq. By characterising immune cell dynamics and key molecular pathways, we sought to provide insights that may guide the development of immune‐targeted therapeutic strategies to promote bone healing and improve clinical outcomes.

## 2 Methods

### 2.1 Study Design

Ethical approval was obtained from the Institutional Review Board of Pusan National University Yangsan Hospital (IRB No. PNUYH‐2022‐033), and all participants provided both verbal and written informed consent for the use of their blood samples.

A total of eight male patients who underwent SA for PSA secondary to displaced intra‐articular calcaneal fractures (DIACFs) between August 2022 and April 2023 were included. All surgeries were performed by a single senior foot and ankle surgeon (SHW). The arthrodesis site was exposed using the sinus tarsi approach [20]. After approaching the arthrodesis site, the remnant cartilage of the posterior facet was removed, and the fresh bone surface was revealed by the removal of sclerotic subchondral bone [20]. Cancellous allograft bone was then placed at the arthrodesis site, and two 6.5‐mm partially threaded cannulated screws were fixed in an angulated configuration [21].

**FIGURE 1:**
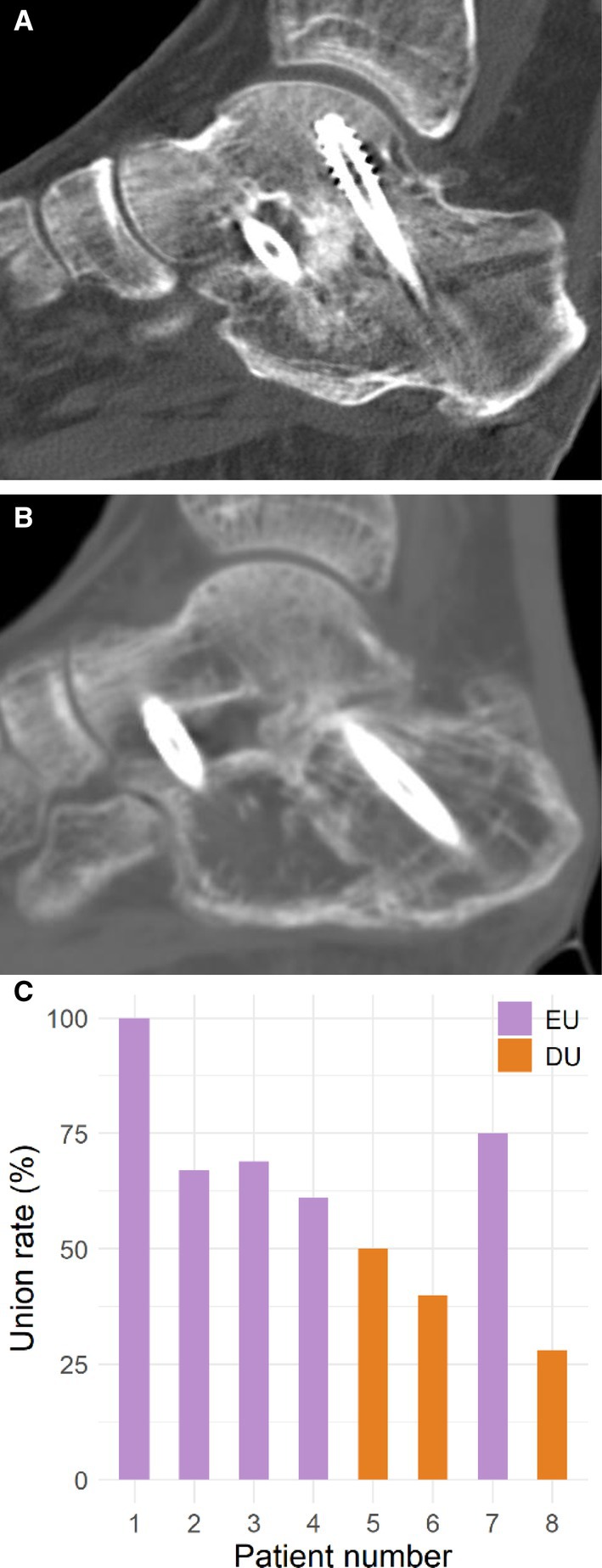
Union rate assessment by computed tomography (CT) scans. The extent of bony union was quantitated on the CT scans. On sagittal cut of the subtalar joint, the width of the total arthrodesis site and the width of the union (defined as bony trabeculation or absence of radiolucent area) were measured on the picture archiving and communication system and recorded. The sum total widths of the union areas were divided by the sum total widths of the arthrodesis site and this ratio was expressed as a percentage of the union rate for SA. (A) Patient number 7, classified EU group with 75% union rate on CT scans at 3 months postoperative (B) Patient number 8, classified DU group with 28% union rate on CT scans at 3 months postoperative (Table S1). (C) Bar graphs to display CT‐based union measurements for all patients. Based on CT scan assessments, patients were categorised into the EU group (blue, ≥ 50% bone union, five patients) and the DU group (yellow, < 50% bone union, three patients). Based on CT scan assessments, patients were categorised into the EU group (≥ 50% bone union, five patients) and the DU group (< 50% bone union, three patients) (Figure 1A–C).

Patients were scheduled for outpatient follow‐up visits at 4, 8 and 10 weeks, and subsequently at 3, 6 and 12 months postoperatively. Bony union was defined according to the following criteria: (1) resolution of heel pain during weight‐bearing; and (2) bony trabeculae involving more than half of the subtalar joint on the standing lateral radiographs. As routine CT scans at every visit were not feasible, all eight patients underwent CT scans at 3 months postoperatively to quantitatively assess the extent of union. On sagittal cuts of the subtalar joint, the width of the total arthrodesis site and the width of the union (defined as bony trabeculation or absence of radiolucent area) were measured on the picture archiving and communication system (PACS) and recorded. The sum total widths of the union areas were divided by the sum total widths of the arthrodesis site, and this ratio was expressed as a percentage of the union rate for SA (Figure 1).

### 2.2 PBMC Collection and Isolation

Venous blood samples were collected by routine venipuncture before surgery and at 3 months postoperatively, using ethylenediaminetetraacetic acid tubes. PBMCs were isolated using SepMate within 30 min of collection. A density gradient medium was introduced into the insert, and the SepMate tube was filled with an equal volume of blood samples diluted in phosphate‐buffered saline (PBS) containing 2% fetal bovine serum (FBS). The samples were then centrifuged at 1200 × g for 10 min at room temperature. The upper layers were carefully collected and washed twice with PBS supplemented with 2% FBS. Subsequently, the tubes were centrifuged at 120 × g for 10 min at room temperature. The resulting PBMCs were frozen and stored at −80°C until future use.

In total, PBMCs were collected from eight patients before surgery, and from seven of them again at 3 months postoperatively. Thus, the final dataset comprised seven paired pre‐/postoperative samples and one unpaired preoperative sample.

### 2.3 Sample Processing for Single‐Cell RNA Sequencing Library

Cryopreserved cell stocks were thawed in 10% Fetal Bovine Serum (FBS)/Dulbecco's Modified Eagle Medium (DMEM) at 37°C. The specimens underwent dual cleansing cycles with cold calcium and magnesium‐free 0.04% Bovine Serum Albumin (BSA)/Phosphate Buffered Saline (PBS), centrifuged at 300 g for 5 min at 4°C. The resulting pellet was gently resuspended in a stain buffer (BD Biosciences, cat no. 554656). Cellular enumeration was performed using a LUNA‐FX7 Automated Fluorescence Cell Counter (Logos Biosystems), stained with Acridine Orange (AO) and Propidium Iodide (PI) staining (Logos Biosystems, cat no. F23001). For sample multiplexing, each sample was labelled with human cell‐specific antibody‐polyadenylated DNA barcodes (BD Biosciences, cat no. 633781). This process involved a 20‐min incubation with the multiplexing antibody at room temperature, followed by a triple wash using stain buffer (BD Biosciences, cat no. 554656). After final cleansing, the samples were gently resuspended in chilled sample buffer (BD Biosciences, cat no. 664887), quantified using the LUNA‐FX7 counter, and subsequently combined.

### 2.4 Single‐Cell RNA Sequencing Library Construction

The BD Rhapsody HT Xpress system was applied for individual cell isolation, adhering to the manufacturer's instructions. A mixture of cells from each sample, suspended in the cold sample buffer, was introduced into the BD Rhapsody 8‐Lane cartridge (BD Biosciences, cat no. 666262). Following cell separation, magnetic beads with cell‐specific barcodes were added to the cartridge. Cells underwent lysis, and mRNA‐bound beads were extracted. The BD Rhapsody cDNA kit (BD Biosciences, cat no. 633773) was utilised to synthesise cDNA and perform Exonuclease I treatment on the captured mRNA beads. scRNA‐seq library construction followed the ‘mRNA Whole Transcriptome Analysis and Sample Tag Library Preparation’ protocol, using the BD Rhapsody Whole Transcriptome Analysis (WTA) amplification kit (BD Biosciences, cat no. 633801). For the WTA library, cDNA underwent a series of processes: random priming and extension (RPE), RPE amplification and index PCR.

### 2.5 Library Sequencing and Quantification

The DNA libraries were quantified using qPCR, adhering to the qPCR Quantification Protocol Guide (KAPA). Quality assessment was performed using the Agilent Technologies 4200 TapeStation. Sequencing was performed on the NovaSeq 6000 platform from Illumina, and the raw FASTQ files were analysed using the BD Rhapsody WTA Analysis Pipeline. Finally, the processed data were aligned to the human reference genome GRCh38.

### 2.6 Preprocessing of Single‐Cell RNA Sequencing Data

The scRNA‐seq data were analysed using the Seurat R package (version 4.2.0) [22]. The barcode‐cell matrices were imported, and the Seurat objects were created using fread and CreateSeuratObject function. Individual samples were identified via the Sample_Tag column in the Sample_Tag_Calls files generated by the SevenBridge platform. After merging objects, a quality control step was implemented to include only high‐quality cells. Cells were retained for further analysis if they met the following criteria: fewer than 20,000 unique molecular identifiers (UMIs), more than 850 and < 5000 expressed genes, and a mitochondrial gene percentage < 20%. This process yielded 49,310 cells from pre‐surgical patients and 45,811 cells from post‐surgical patients for subsequent analysis. The preprocessed scRNA‐seq count matrix for mouse fracture model was obtained from the Gene Expression Omnibus (GEO) database (accession number GSE132884). This dataset comprises a total of 15,033 cells. Both datasets were normalised using the SCTransform method, and 3000 highly variable features were selected. Principal component analysis (PCA) was then conducted on each individual object. Subsequently, integration of all objects was achieved using anchors, which were chosen by the FindIntegrationAnchors function.

### 2.7 Dimensionality Reduction and Clustering

PCA was performed using 50 PCs on the integrated object. To determine the optimal number of PCs for further analysis, we employed a cumulative variance explained cutoff of 80%. The first 32 PCs were selected and applied to Uniform Manifold Approximation and Projection (UMAP) embedding. We found the k‐nearest neighbours using FindNeighbors and identified cell populations by constructing a Shared Nearest Neighbour (SNN) graph based on the Louvain algorithm.

### 2.8 Identification of Differentially Expressed Genes and Gene Ontology Analysis

The Seurat wrapper FindMarkers function was applied to identify Differentially Expressed Genes (DEGs) between Early Union (EU) and Delayed Union (DU) groups. This process was iteratively performed for each cell type. The minimum log fold‐change and proportion threshold were set at 0.25% and 10% to mitigate the inclusion of spurious transcripts. Statistical significance for DEGs was determined using a Bonferroni‐corrected adjusted *p*‐value threshold of 0.05. For the functional annotation of DEGs, we used the clusterProfiler package [23]. Gene Ontology (GO) analysis was performed, focusing on the biological process subontology. The org.Hs.eg.db database was utilised as the reference for human gene annotations. The functional terms with fewer than three enriched genes were excluded, and only terms with *p*‐values < 0.05 were included.

### 2.9 Scoring of Biological Signatures

We assigned each cell a single biological score calculated based on multiple genes through the Seurat wrapper AddModuleScore function. The gene lists related to biological functions were obtained from MSigDB (https://www.gsea‐msigdb.org/gsea/msigdb) [24]. Gene sets for GO:0050766 (positive regulation of phagocytosis), GO:0019886 (antigen processing and presentation of endogenous antigens) and GO:0032816 (positive regulation of natural killer cell activation) were retrieved, and the scores were computed based on their expression.

### 2.10 Cell–Cell Communication Analysis

Cellular communication inference was performed using the CellChat package (v. 1.6.1) [25]. Cells from each pre‐surgical EU and DU group were extracted, and intercellular communication analysis was conducted for each group. CellChatDB.human was used as a ligand–receptor interaction repository. Preprocessing, including identification of overexpressed genes, overexpressed interactions and data projection, was performed using default parameters. Subsequently, communication probability and cellular communication networks were calculated. Cell groups with fewer than 10 cells were excluded from estimating cell–cell communication. To compare the probability of communication networks between pre‐surgical EU and DU groups, the rankNet function was used. To visualise the network of signalling pathways, the netVisual_circle function and the patchwork package were used.

### 2.11 Statistical Analysis

To determine significant differences between two groups, we performed non‐parametric tests. The Wilcoxon rank sum test was used for scRNA‐seq–derived comparisons, and the Mann–Whitney *U* test was specifically applied to assess demographic differences such as age between the EU and DU groups. The *p*‐values are denoted as follows: *p < 0.05, **p < 0.01, ***p < 0.001 and ****p < 0.0001.

## 3 Results

### 3.1 Single‐Cell RNA Sequencing Landscape of PBMCs in Pre‐Surgical Patients

**FIGURE 2:**
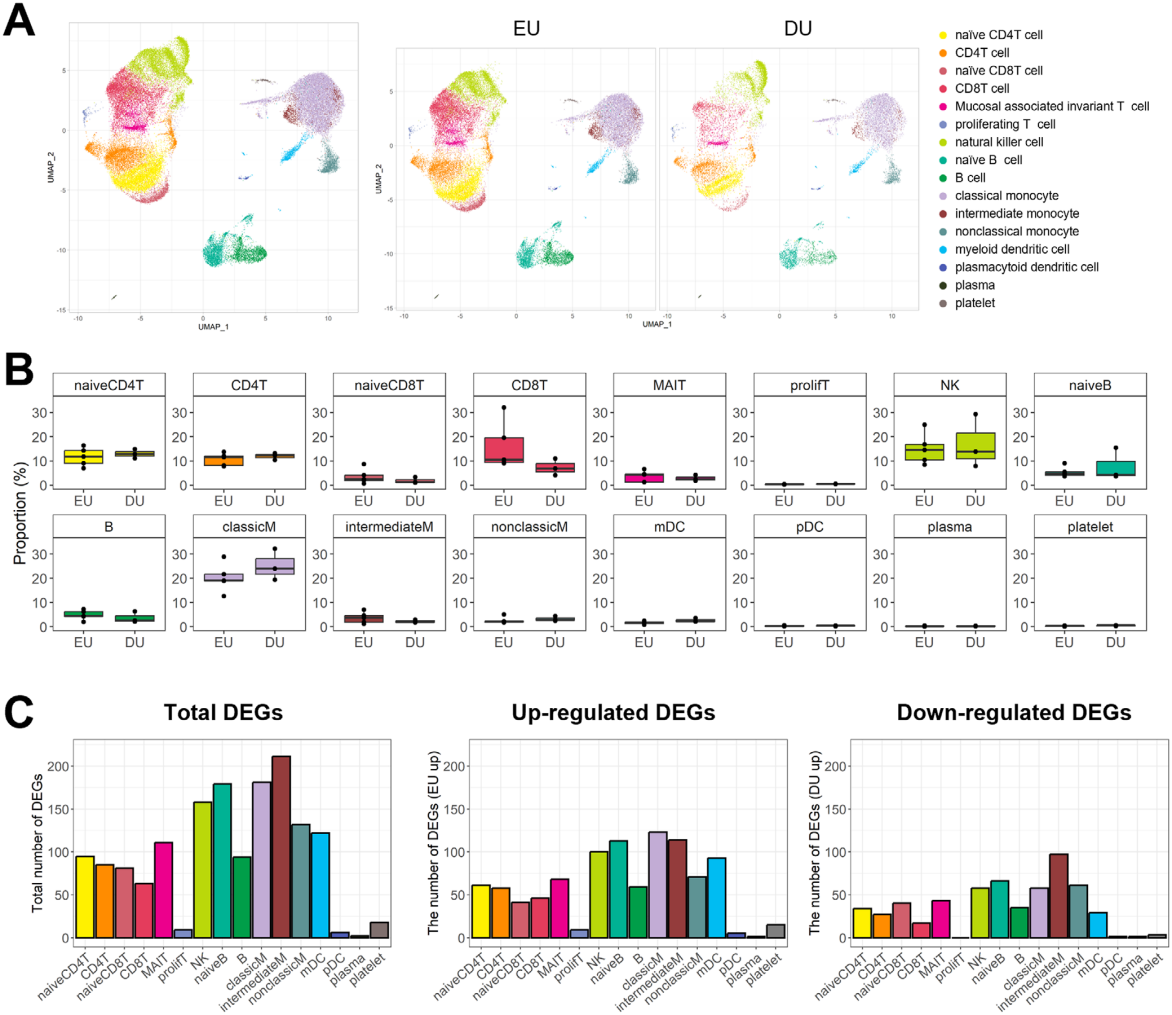
Cellular composition and gene expression differences revealed by single‐cell analysis of PBMCs from patients with subtalar arthrodesis prior to surgery. (A) UMAP of PBMCs obtained from patients prior to subtalar arthrodesis. Cellular identities from eight patients were shown in the left panel, whereas cellular populations from EU and DU groups were segregated in the right panel. (B) Fraction of cell identities stratified by union groups. *p*‐values are not displayed as none reached statistical significance at the 0.05 threshold. (C) The number of DEGs comparing pre‐surgical EU and DU groups for each cell type. The first panel shows the total number of detected DEGs for each cell type, and the second and third panels illustrate the number of up‐regulated DEGs and down‐regulated DEGs in the EU group compared to the DU group, respectively.

Our study analysed PBMC samples obtained from eight patients with PSA prior to surgery. Based on CT scan assessments, patients were categorised into the EU group (≥ 50% bone union, five patients) and the DU group (< 50% bone union, three patients) (Figure 1A–C). Among these, seven patients provided paired pre‐ and postoperative samples collected 3 months after surgery, while one patient had only a preoperative sample available. In total, eight preoperative and seven postoperative datasets were included in the analysis. The clinical characteristics of the patients are summarised in Table S1. The mean age of patients in the EU group was 54.8 years (range: 26–72 years), and that of patients in the DU group was 64.7 years (range: 50–78 years). Although the mean age of patients in the DU group appeared to be older, the difference was not statistically significant (*p* = 0.571). Following preprocessing of the scRNA‐seq data, we obtained transcriptional profiles encompassing 24,444 genes across 49,310 cells. Clustering analysis revealed 28 distinct cellular clusters which were annotated into 16 immune cell types, including classical and intermediate monocytes (classicM, intermediateM), natural killer (NK) cells, CD4^+^ and CD8^+^ T cell subsets (naïve, effector and proliferating), B cell subsets (naïve and plasma), and dendritic cells (myeloid [mDC] and plasmacytoid [pDC]) (Figure 2A, Figure S1A,B). Upon dimensional reduction, the cells exhibited a broadly homogeneous distribution across both EU and DU groups. Although the overall abundance of most immune cell types was comparable between the groups, intra‐population transcriptional heterogeneity was evident within certain populations, particularly NK cells, reflecting functional diversity rather than numerical differences. NK cells displayed distinct transcriptional programs, with subsets showing cytotoxic effector signatures in the DU group and cytokine/chemokine‐related regulatory profiles in the EU group, suggesting divergent activation states rather than compositional imbalance, as explored in subsequent analyses.

We first examined cellular composition at the individual patient level (Figure 2B). classicM were the most abundant cell type across all patients, followed by NK cells and CD8^+^ T cells. Though there were no significant proportional differences between EU and DU groups, there was substantial inter‐patient heterogeneity, particularly in the proportions of NK cells (8.5%–29.3%) and CD8^+^ T cells (4.2%–32.1%), indicating marked variability among individuals. Despite this compositional diversity, there was a lack of a consistent pattern associated with bone union status (Figure S1C).

Given the observed variability in the cell populations, we hypothesized that functional changes in immune cells, rather than their abundance, might be more relevant to bone healing. Thus, we first investigated the number of differentially expressed genes (DEGs) across cell compartments (Figure 2C). The analysis revealed pronounced transcriptional alterations, most prominently detected within monocytes, particularly classicM, which play a crucial role in innate immunity. Additionally, significant gene expression changes were detected in naïve B cells, NK cells and myeloid dendritic cells. Monocytes and NK cells were prioritised for further investigation due to their established involvement in orchestrating the initial inflammatory response for bone repair [26, 27].

### 3.2 Functional and Molecular Characteristics of Classical Monocyte and Intermediate Monocyte in Pre‐Surgical EU and DU Groups

**FIGURE 3:**
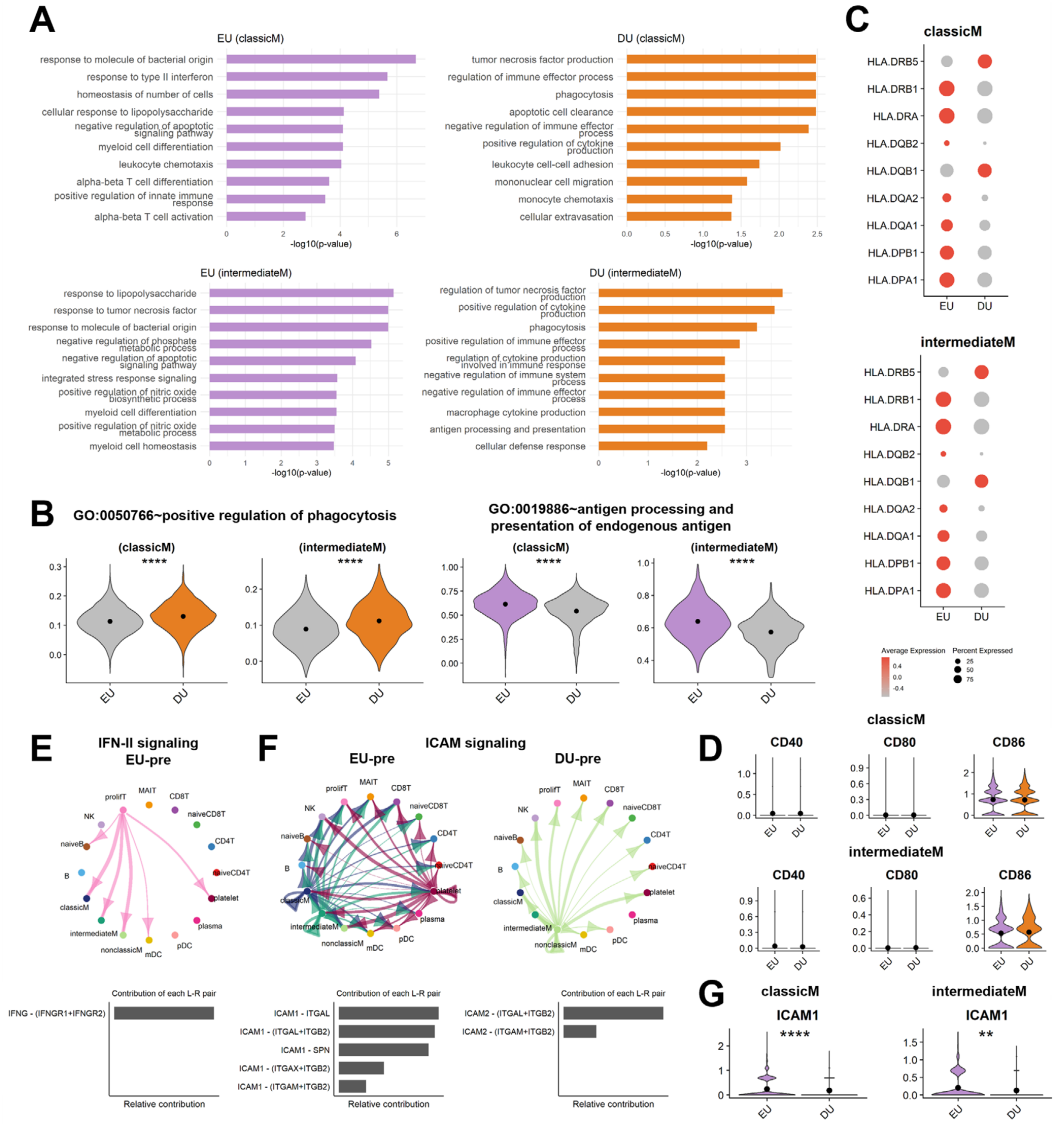
Functional and molecular properties of classicM and IntermediateM in pre‐surgical EU and DU groups. (A) GO terms observed in classicM and intermediateM. The biological functions associated with up‐regulated DEGs in EU and DU groups prior to surgical intervention are shown. The upper panel demonstrates the GO terms observed in classicM and the lower panel exhibits the GO terms in intermediateM. (B) The module score of phagocytosis and antigen processing and presentation. The scores were calculated by gene sets from ‘GO:0050766~positive regulation of phagocytosis’ and ‘GO:0019886~antigen processing and presentation of endogenous antigen’. Comparison of these scores were performed in classicM and intermediateM, respectively. The *p*‐values are as follows: **p < 0.01, ****p < 0.0001. (C) Expression levels of MHC class‐II encoding genes prior to surgery. The dot plots display the gene expression profiles of HLA genes between EU and DU groups in classicM and intermediateM. The colour intensity of dot corresponds to the magnitude of gene expression, while the size of the dot represents the percentage of cells expressing the gene. (D) The violin plots for co‐stimulatory molecules, CD40, CD80 and CD86, in classicM and intermediateM. (E) The cell–cell interaction network of the IFN‐II signalling pathway. The source cells, from which the arrows originate, correspond to the signal‐sending cells, whereas the target cells, at which the arrows terminate, represent the signal‐receiving cells. The bar plot in the lower section depicts the ligand‐receptor pairs involved in the signalling. (F) The cell–cell interaction network of the ICAM signalling pathway. The first network exhibits the interactions generated in the EU group, while the second network represents those in the DU group. (G) The violin plots for ICAM1 expression level in classicM and intermediateM.

In the preoperative EU group, classicM and intermediateM exhibited transcriptional upregulation in pathways related to the response to molecules of bacterial origin, IFN‐II (IFN‐γ) response, lipopolysaccharide response, alpha‐beta T cell differentiation and alpha‐beta T cell activation (Figure 3A). In the DU group, classicM and intermediateM showed increased expression of genes related to tumour necrosis factor (TNF) production and phagocytosis (Figure 3A). Since both classicM and intermediateM are involved in phagocytosis and antigen presentation, we compared their transcriptional profiles between the EU and DU group. Whereas the DU group exhibited elevated phagocytic activity, antigen processing and presentation of endogenous antigens were more prominent in the EU group (Figure 3B). As key molecules involved in antigen presentation, the transcript levels of all human leukocyte antigen‐DR subunit‐encoding genes were elevated in the EU group (Figure 3C). Comparable expression of costimulatory molecules CD40, CD80 and CD86 further supports the importance of HLA in the role of antigen presentation between the EU and DU groups across both classicM and intermediateM (Figure 3D).

To explore intercellular communication, we analysed cell–cell interactions. In the EU group, the IFN‐γ and ICAM signalling were identified as key pathways (Figure S2). Notably, IFN‐II signalling was detected exclusively in the EU group and was absent in the DU group (Figure 3E). Although ICAM signalling was observed in both groups, its activity was markedly higher in the EU group (Figure 3F). Additionally, in the EU group, classicM and intermediateM interacted through ICAM1, whereas in the DU group, non‐classicM engaged through ICAM2. Expression of ICAM1 was significantly higher in both classicM and intermediateM in the EU group compared to the DU group (Figure 3G).

### 3.3 Functional and Molecular Characteristics of NK Cells in Pre‐Surgical EU and DU Groups

**FIGURE 4:**
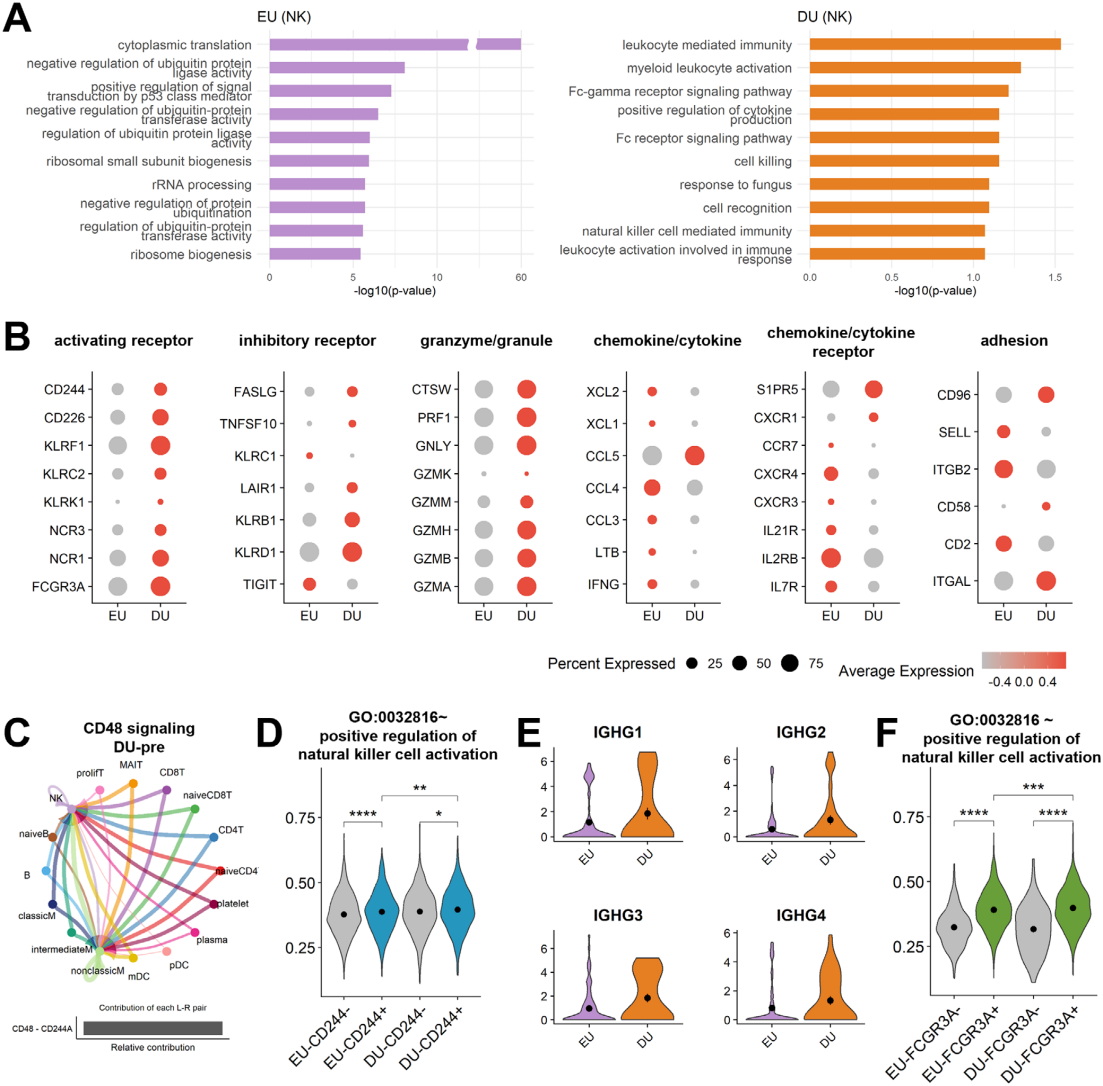
Distinct functional characteristics of NK cells between EU and DU groups prior to surgical intervention. (A) GO terms observed in NK cells. The left panel describes the upregulated biological processes in pre‐surgical EU group, while the right panel illustrates those in pre‐surgical DU group. (B) Expression levels of NK cell‐related molecules. The categories of molecules include activating receptor, inhibitory receptor, granzyme/granule, chemokine/cytokine, chemokine/cytokine receptor and adhesion molecule. (C) The cell–cell interaction network of the CD48 signalling pathway. The bar plot in the lower section depicts the ligand‐receptor pairs involved in the signalling. (D) The module score of NK cell activation according to CD244 expression. The scores were calculated by gene sets from ‘GO:0032816~positive regulation of natural killer cell activation’. The NK cell groups are stratified based on CD244 expression status (CD244+ or CD244‐) within the EU and DU groups. The *p*‐values are as follows: *p < 0.05, **p < 0.01, ***p < 0.001, ****p < 0.0001. (E) The violin plots showing the expression levels of the IgH subclasses in plasma cells. (F) The module score of NK cell activation depending on FCGR3A expression. The NK cells are grouped based on FCGR3A expression status (FCGR3A+ or FCGR3A−) within the pre‐surgical EU and DU groups.

Next, we investigated the functional differences between the EU and DU preoperative groups in NK cells. In the EU group, pathways related to cytoplasmic translation and ribosome biogenesis were upregulated, suggesting that NK cells in the EU group were oriented toward biosynthetic and cytokine/chemokine‐mediated immune regulation that may support tissue repair. In contrast, NK cells in the DU group exhibited increased activation of the Fc‐gamma receptor signalling pathway and enhanced cell killing, indicating a pro‐inflammatory and cytotoxic state that may contribute to delayed healing (Figure 4A). Consistent with these functional differences, chemokines, cytokines and their receptors were highly expressed in the EU group (Figure 4B), whereas activating receptors and granzyme/granule‐related genes were significantly upregulated in the DU group.

Cell–cell interaction analysis revealed that the CD48 signalling pathway was uniquely present in the DU group (Figure S2). Notably, NK cells displayed a central role in receiving the CD48 signal (Figure 4C). Since CD244 is activated upon binding to CD48, CD244‐expressing NK cells can be effectively stimulated, leading to enhanced cytotoxicity [28]. Consequently, NK cell activation was significantly elevated in CD244‐expressing NK cells, with the highest levels observed in the DU group (Figure 4D).

In alignment with these findings, GO analysis of the DU group revealed increased enrichment of gene sets involved in the FCGR3A signalling pathway and cytotoxic molecules. This suggests a potential role for antibody‐dependent cellular cytotoxicity (ADCC) mediated by IgG recognition. While IgG expression in plasma cells did not reach statistical significance, it was consistently higher in the DU group (Figure 4E). To further explore FCGR3A‐driven NK cell activation, we stratified NK cells based on FCGR3A expression (Figure 4F). As a result, FCGR3A‐expressing NK cells exhibited significantly higher activation in both the DU group and EU groups, with the highest activation in the DU group. These findings indicate that NK cell activation is influenced by multiple signalling axes, and bone union efficiency may be modulated through both CD244‐ and FCGR3A‐dependent pathways.

### 3.4 Validation of Functional and Molecular Characteristics in Postoperative Patients

**FIGURE 5:**
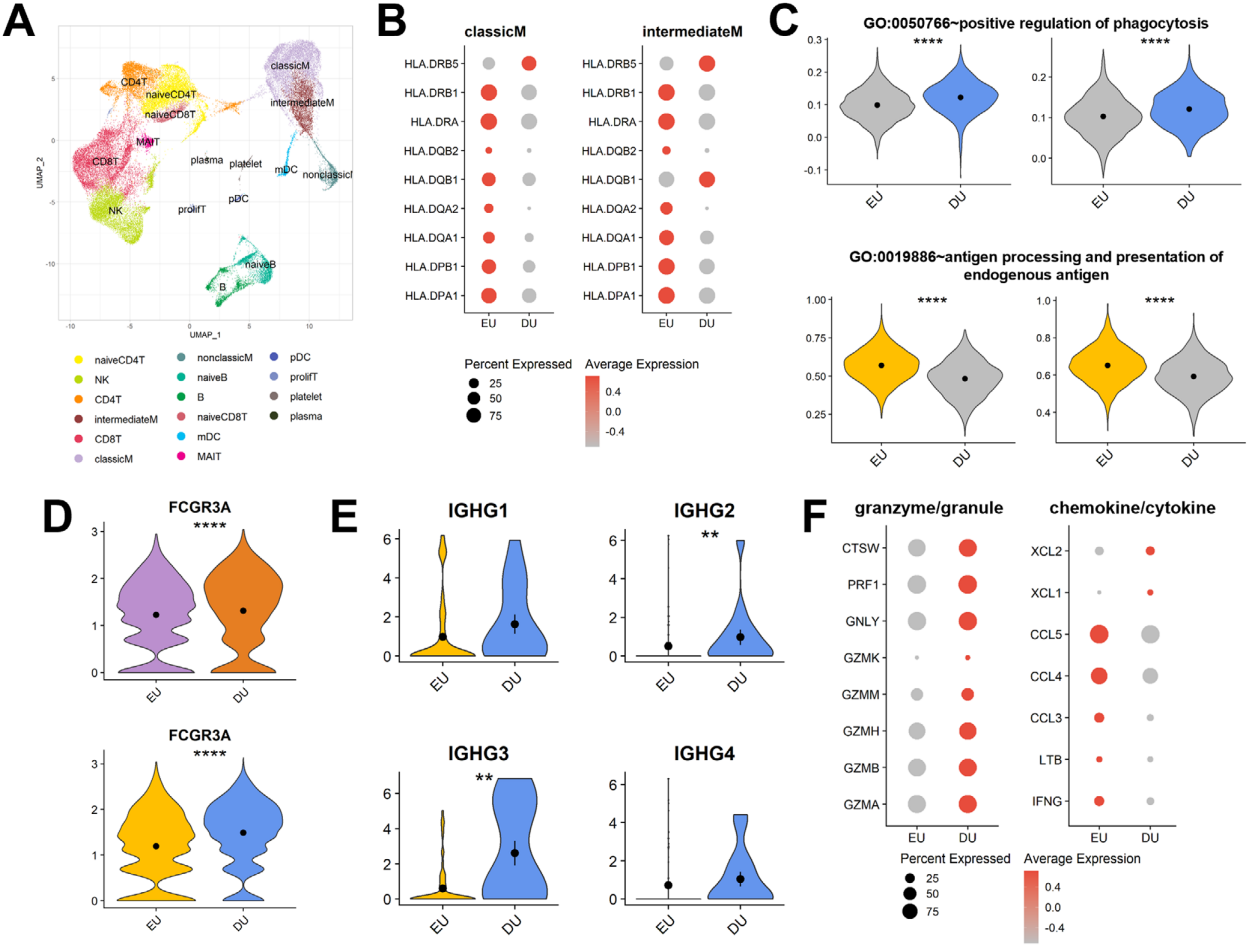
Identical attributes in monocyte and NK cells between EU and DU groups after surgery. (A) UMAP visualisation of PBMCs collected from the same patients after subtalar arthrodesis. (B) Expression levels of HLA genes in postoperative patients. The HLA genes are compared between EU and DU groups in classicM and intermediateM. (C) The module score of phagocytosis and antigen processing and presentation in classicM and intermediateM. The comparison was conducted within patients who underwent surgery. The *p*‐values are as follows: **p < 0.01, ****p < 0.0001. (D) The FCGR3A expression level in NK cells between EU and DU groups. The upper panel compares expression levels in preoperative patients, while the lower panel compares them in postoperative patients. (E) The IgG subclasses expression level in plasma cells between EU and DU groups after surgery. (F) The dot plot displaying the transcriptional levels of granzymes/granules and chemokines/cytokines in NK cells from patients who underwent subtalar arthrodesis.

To determine whether the immune signatures associated with bone union velocity were intrinsic and sustained over time, we conducted a longitudinal assessment of immune profiles in the same patient cohort 3 months after surgery. scRNA‐seq was performed on postoperative PBMCs, with one patient excluded due to the absence of a postoperative sample. After quality control and preprocessing, we obtained transcriptional profiles of 25,036 genes across 45,811 cells. A total of 16 immune cell types were identified from 28 clusters using canonical marker genes (Figure 5A, Figure S3).

Among monocyte subsets, HLA subunit–encoding genes were more highly expressed in the EU group than in the DU group (Figure 5B). Aligning with prior findings, phagocytosis was markedly increased in both classicM and intermediateM of the DU group, whereas antigen processing and presentation of endogenous antigens were more pronounced in the EU group (Figure 5C). Notably, FCGR3A expression in NK cells remained persistently higher in the DU group across preoperative and postoperative time points (Figure 5D). The DU group demonstrated heightened IgG production, with IGHG2 and IGHG3 subtypes showing statistically significant increases (Figure 5E). Transcriptional comparison further revealed that chemokines and cytokine‐related genes were highly expressed in the EU group, while granzyme/granule‐related genes were significantly upregulated in the DU group (Figure 5F). Collectively, these longitudinal findings indicate that distinct immune activation patterns are maintained across time points, suggesting that baseline immune imbalances contribute to differences in fracture healing rates.

### 3.5 Verification Through Immunological Dynamics According to Bone Healing Stages

**FIGURE 6:**
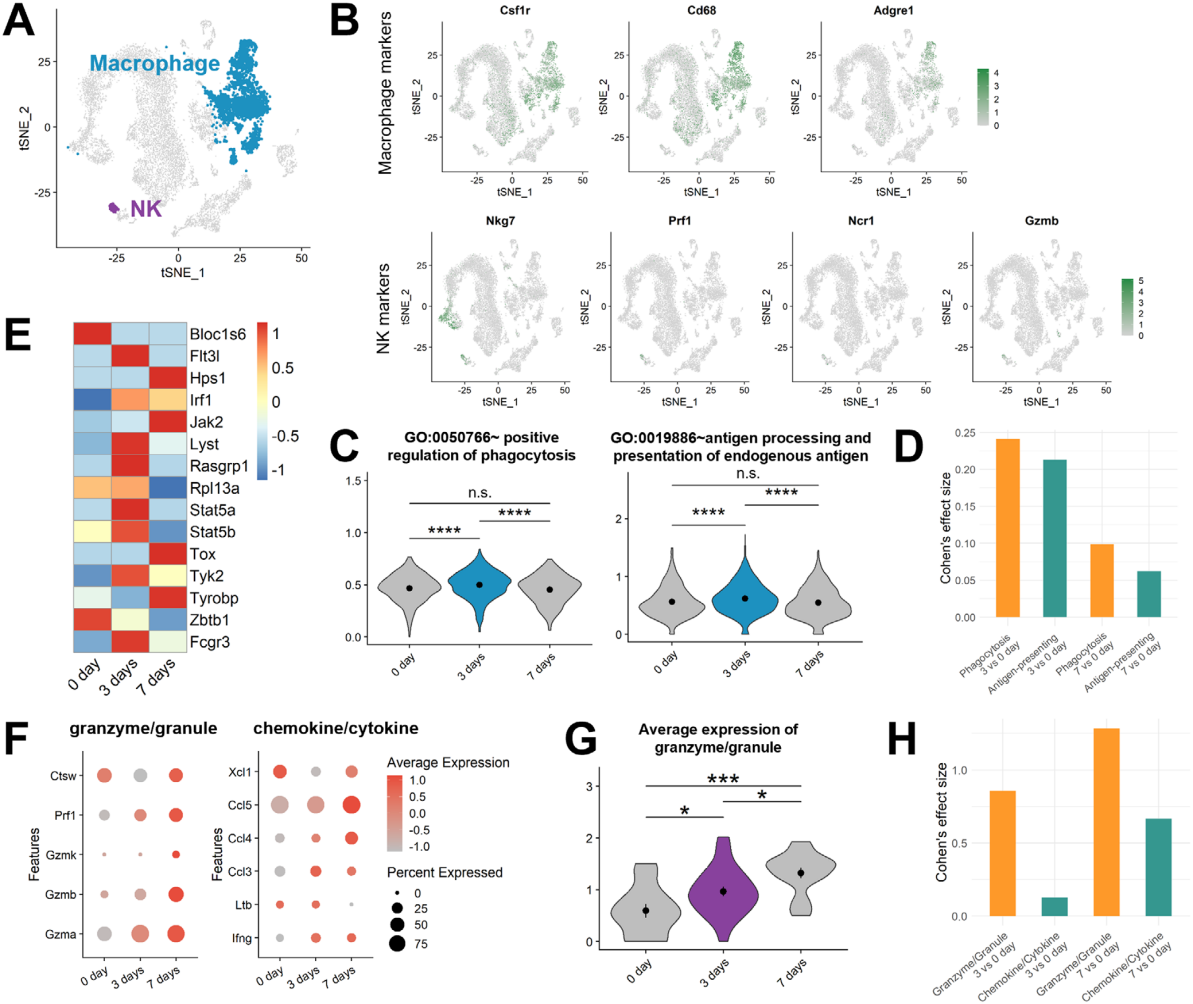
Dynamic changes in macrophage and NK cell populations during mouse fracture healing. (A) UMAP visualisation of scRNA‐seq data from mouse fracture tissue. Cell clusters corresponding to macrophages and NK cells are highlighted in blue and purple, respectively. (B) Feature plots illustrating the expression intensity of key gene markers used for the identification of Macrophages (top panel) and NK cells (bottom panel). The colour gradient indicates the relative expression level of the respective genes across the cell populations. (C) The module score analysis comparing the pathway activity of phagocytosis (left) and antigen processing and presentation (right) within macrophages population across different time points. The *p*‐values are as follows: *p < 0.05, ***p < 0.001, ****p < 0.0001, *p* > 0.05: Not significant (n.s.). (D) Cohen's effect size for phagocytosis (golden yellow) and antigen processing and presentation (green). Effect sizes at 3 and 7 days were compared to the control (day 0) within macrophage population. (E) Heatmap depicting the expression profile of genes involved in ‘GO:0032816~positive regulation of natural killer cell activation’ across the NK cell population, categorised by the fracture time point. (F) Dot plot displaying the transcriptional levels of genes encoding granzymes and granules (left) and chemokines and cytokines (right) in NK cells isolated from fractured mouse tissue. (G) Violin plot illustrating the average transcriptional levels of granzyme/granule within NK cells, comparing different time points in the mouse fracture healing model. (H) Cohen's effect size for granzyme/granule (golden yellow) and chemokine/cytokine (green). Effect sizes at 3 and 7 days were compared to the control (day 0) within NK cell population.

To investigate whether the baseline immune characteristics identified in circulation influence bone healing velocity, we examined their temporal changes within fractured tissue during distinct healing stages. We analysed single‐cell RNA sequencing (scRNA‐seq) data from a mouse fracture model, which included macrophages and NK cells (Figure 6A,B) [18]. Fractured bone tissues were collected at three time points: Day 0 (control), Day 3 (initial inflammatory phase) and Day 7 (late inflammatory/callus formation phase). First, the antigen‐presenting ability and phagocytosis of macrophages were highest on Day 3, corresponding to the hematoma stage (Figure 6C). Specifically, the effect size, which represents the change in gene expression relative to the control, showed a greater change in phagocytosis compared to antigen presentation at both Days 3 and 7 (Figure 6D). This suggests a substantial change in phagocytosis during the inflammatory phase. Meanwhile, NK cells exhibited high activity at Days 3 and 7, and the expression of granzymes/granules and chemokines/cytokines generally increased throughout the inflammatory phase (Figure 6E,F). The change in granzymes/granules progressively increased toward the later stages of inflammation (Figure 6G, Figure S4). Furthermore, the increase in granzymes/granules relative to the normal state was distinctly higher than the increase in chemokines/cytokines across the entire inflammatory phase. Collectively, these results indicate that the changes in macrophage phagocytosis and NK cell granzymes/granules contribute more significantly to the inflammation of the hematoma stage. Therefore, the circulatory baseline characteristics of DU, where both of these changes were notably higher compared to EU, indicate a clear predisposition toward a pro‐inflammatory state, thereby disrupting the immunological balance.

## 4 Discussion

In this study, we performed scRNA‐seq of PBMCs from patients who underwent SA to investigate the systemic osteoimmunological response during bone healing. Our findings revealed distinct immune cell transcriptional profiles between the EU and DU groups. Specifically, monocyte subtypes displayed divergent immunological functions, and NK cells in the DU group exhibited increased cytotoxic activity influenced by multiple pathways. Because these functional disparities persisted postoperatively, we suggest that systemic immune cell function, rather than compositional changes alone, plays a critical role in bone healing.

Innate immunity plays a crucial role in both tissue repair and pathogen defence through various mechanisms, including complement activation, immune cell recruitment, phagocytosis, infected cell elimination and adaptive immune activation via antigen presentation [29]. Innate immune cells such as monocytes and NK cells contribute to bone healing by phagocytosing necrotic cells and fibrin thrombi [26]. Additionally, they regulate the healing process through cytokine secretion, which directly or indirectly influences B and T cell activation [26, 27]. Although few studies have directly elucidated the mechanisms by which monocytes and NK cells stimulate adaptive immune cells in bone immunology, the essential role of innate cells in activating adaptive responses is well recognised [30, 31]. In this study, classicM and intermediateM in the EU group exhibited increased expression of antigen‐processing and presentation genes, whereas DU monocytes showed an enhanced phagocytic response (Figure 3A,B). Notably, IFN‐γ function was activated exclusively in the EU group (Figure 3E,F). IFN‐γ plays a crucial role in osteoimmunology by enhancing macrophage activation and antigen presentation [32]. Monocytes stimulated by IFN‐γ differentiate into macrophages or Dendritic Cells (DCs), enhancing their antigen‐presenting capabilities [33]. Accordingly, IFN‐γ upregulates ICAM‐1 expression [34], as observed in the ICAM signalling pathway of the EU group, which is known to facilitate T cell activation and migration to target tissues (Figure 3E–G) [35]. Therefore, a functional decline in antigen presentation capacity may impair adaptive immune activation, including B‐ and T‐cell responses, and ultimately delay bone repair.

NK cells in the DU group exhibited increased cytotoxic activity, characterised by the upregulation of Fc‐gamma receptor signalling and ADCC pathways. Evaluation of NK cell activation via the CD48‐CD244 intercellular pathway revealed higher activation levels in the DU group (Figure 4C,D) [36]. In addition, FCGR3A, a receptor capable of activating NK cells independently of costimulatory signals, was persistently overexpressed in the DU group across time points (Figure 4E,F). In contrast, NK cells in the EU group expressed higher levels of chemokines, cytokines and their receptors (Figure 4A). The upregulated chemokines and cytokines in the EU group—XCR1, CCL3, CCL4 and IFN‐γ—are known mediators of antigen uptake and the induction of both innate and adaptive cytotoxic immunity [37, 38, 39]. These findings suggest that the EU group may possess an intrinsic advantage in initiating immune cascades, such as immune cell recruitment for debris clearance, rather than relying solely on direct cytotoxicity.

Taken together, these data indicate that DU patients maintain a persistently pro‐inflammatory immune state dominated by phagocytic and cytotoxic activity, which may delay the resolution phase of inflammation and impede bone repair. Conversely, EU patients exhibit an earlier transition toward antigen presentation and chemokine‐mediated immune coordination, supporting controlled inflammatory resolution and more efficient bone union [27, 40].

Although our analysis focused on circulating immune cells, recent single‐cell transcriptomic studies have demonstrated that macrophages, osteoclasts and osteoblast‐lineage cells closely interact within the bone microenvironment to coordinate bone remodelling and repair [17, 41, 42]. In particular, macrophage–osteoblast and osteoclast–osteoblast signalling loops regulate the balance between inflammation, resorption and new bone formation [17, 41, 42]. These findings suggest that systemic immune polarisation observed in our study, such as enhanced phagocytic and cytotoxic activity in the delayed union group, may influence local osteoimmune interactions at the arthrodesis site, thereby affecting bone fusion outcomes.

We further analysed PBMC samples from seven postoperative patients with SA. In the EU group, monocytes and NK cells maintained elevated antigen‐presentation activity, whereas the DU group continued to show enhanced phagocytic and cytotoxic features (Figure 5B–F). This suggests that the systemic immune milieu before and after surgery remains largely consistent, indicating that baseline immune polarisation may strongly influence healing outcomes. Previous studies have shown that chronic inflammation and immunosenescence can alter cytokine signalling and immune competence, thereby delaying bone regeneration. Notably, research on immunoaging—integrating age‐associated and inflammaging‐related changes—has shown that younger, immunologically less‐experienced individuals develop a more stable callus, whereas immunoaged individuals exhibit delayed callus formation and structural instability [40]. Considering the immune disparities between EU and DU groups, broader determinants such as aging and chronic inflammation must be recognised. Therefore, monitoring baseline immune status in high‐risk patients, such as the elderly or those with osteoporosis, may enable early identification of poor healers, allowing immunomodulatory or supportive interventions to promote optimal recovery.

This study has several limitations. First, PBMC samples were cryopreserved to ensure the selection of individuals who met the subject criteria. However, cryopreservation may have affected the integrity of certain cell types. While T cells from cryopreserved PBMCs have been shown to recover and maintain viability over the long term, the number of monocytes is often reduced compared to freshly isolated PBMCs [43]. Therefore, validation using freshly isolated samples is warranted.

Second, this study focused on the transcriptome of PBMCs, which exclude neutrophils. Neutrophils are first responders during early bone healing [26, 27, 44, 45]. They are the first immune cells to infiltrate the injury site and secrete cytokines and chemokines that recruit and activate monocytes and macrophages, thereby shaping the subsequent immune cascade [26, 44, 45]. Including neutrophil transcriptomic data would further clarify early immune activation and its regulatory effects on monocytes and NK cells [27, 45]. Consequently, the immune landscape captured in this study may not fully represent the entire cellular dynamics of bone healing.

Third, the study primarily relies on scRNA‐seq to infer immune cell function but does not include in vitro or in vivo functional assays to directly validate differences in antigen presentation, phagocytosis and cytotoxicity. Experimental approaches such as flow cytometry, cytokine assays, or knockdown studies could provide additional functional validation and strengthen the conclusions. Additionally, potential confounding factors such as age, sex, comorbidities (e.g., diabetes, osteoporosis), medication use (e.g., corticosteroids, immunosuppressants) and lifestyle factors (e.g., smoking, diet, physical activity) were not extensively explored in relation to immune status and bone healing. These variables could significantly influence immune responses and outcomes, highlighting the need for further studies with comprehensive patient stratification. Finally, this study investigates systemic immune responses using PBMCs rather than local immune responses at the arthrodesis site. Because bone‐resident macrophages, osteoclasts and periosteal cells play key roles in tissue remodelling, future research should incorporate local tissue or synovial analyses to provide a more comprehensive view of osteoimmunology in bone repair.

This study provides novel insights into systemic osteoimmunological mechanisms following SA. Our results reveal divergent functional polarisation of monocytes and NK cells as major determinants of bone repair efficiency, and highlight potential immunotherapeutic targets to enhance bone healing. Future research should focus on developing temporally targeted immunomodulatory strategies to optimise bone union, particularly in high‐risk populations.

## Author Contributions

**Hansong Lee:** data curation, formal analysis, investigation, and writing – original draft preparation, and funding acquisition. **Kihun Kim:** data curation, formal analysis, investigation, and writing – original draft preparation. **Tae Sik Goh:** methodology, software, supervision. **Suk‐Woong Kang:** validation, supervision. **Jung Yun Bae:** validation, supervision, writing – reviewing and editing. **Su‐Yeon Cho:** data curation, formal analysis. **Yujin Kwon:** data curation, formal analysis. **Won Kyu Kim:** validation, supervision, writing – reviewing and editing. **Jin‐Woo Kim:** validation, supervision, writing – reviewing and editing. **Yun Hak Kim:** conceptualization, data curation, formal analysis, funding acquisition, writing – reviewing and editing. **Seung Hun Woo:** conceptualization, data curation, formal analysis, writing – reviewing and editing. All authors have read and agreed to the published version of the manuscript.

## Funding

This research was supported by the Basic Science Research Program through the National Research Foundation of Korea (NRF) funded by the Ministry of Education (RS‐2023‐00273667). This research was supported by the HRD Program for Industrial Innovation through the Korea Institute for Advancement Technology (KIAT) funded by the Ministry of Trade, Industry and Energy (RS‐2025‐02214034). This research was supported by a grant of the Korea Health Technology R&D Project through the Korea Health Industry Development Institute (KHIDI), funded by the Ministry of Health &Welfare, Republic of Korea (RS‐2025‐02223691). This work was supported by KREONET. This study was supported by the Research Institute for Convergence of Biomedical Science and Technology, Pusan National University Yangsan Hospital (30‐2022‐020). This study was supported by a fund from the research program of the Korea Medical Institute (KMI) in 2022 (KMI20220154). The funders played no role in the study design, data collection, analysis, decision to publish, or manuscript preparation.

## Ethics Statement

Both verbal and written informed consent was given by the patients for the use of their blood samples. Institutional review board approval was achieved prior to commencing this study (IRB No. PNUYH‐2022‐033).

## Consent

The authors have nothing to report.

## Conflicts of Interest

The authors declare no conflicts of interest.

## Supporting information


**Figure S1:** Annotation of cell clusters obtained from preoperative patients.
**Figure S2:** Intensity of intercellular signalling in preoperative patients.
**Figure S3:** Identification of cell identities obtained from postoperative patients.
**Figure S4:** Violin plot showing the average transcriptional levels of granzyme/granule within NK cells. The *p*‐values are as follows: • < 0.1, *p* > 0.1: not significant (n.s.).

**Table S1:** Clinical profiles of subjects.

## Data Availability

The data that support the findings of this study are available from the corresponding author upon reasonable request.
