# Supplementary material for: Deciphering the Osteoimmune Landscape in Subtalar Arthrodesis: A Single‐Cell RNA Sequencing Approach
Source: J Cell Mol Med. 2025 Dec 11;29(23):e70980. doi: 10.1111/jcmm.70980 (PMC12698338; doi:10.1111/jcmm.70980)
Supplement: Supplementary file 1 — Figure S1: Annotation of cell clusters obtained from preoperative patients. Figure S2: Intensity of intercellular signalling in preoperative patients. Figure S3: Identification of cell identities obtained from postoperative patients. Figure S4: Violin plot showing the average transcriptional levels of granzyme/granule within NK cells. The p‐values are as follows: • < 0.1, p > 0.1: not significant (n.s.). [file JCMM-29-e70980-s002.docx]

**
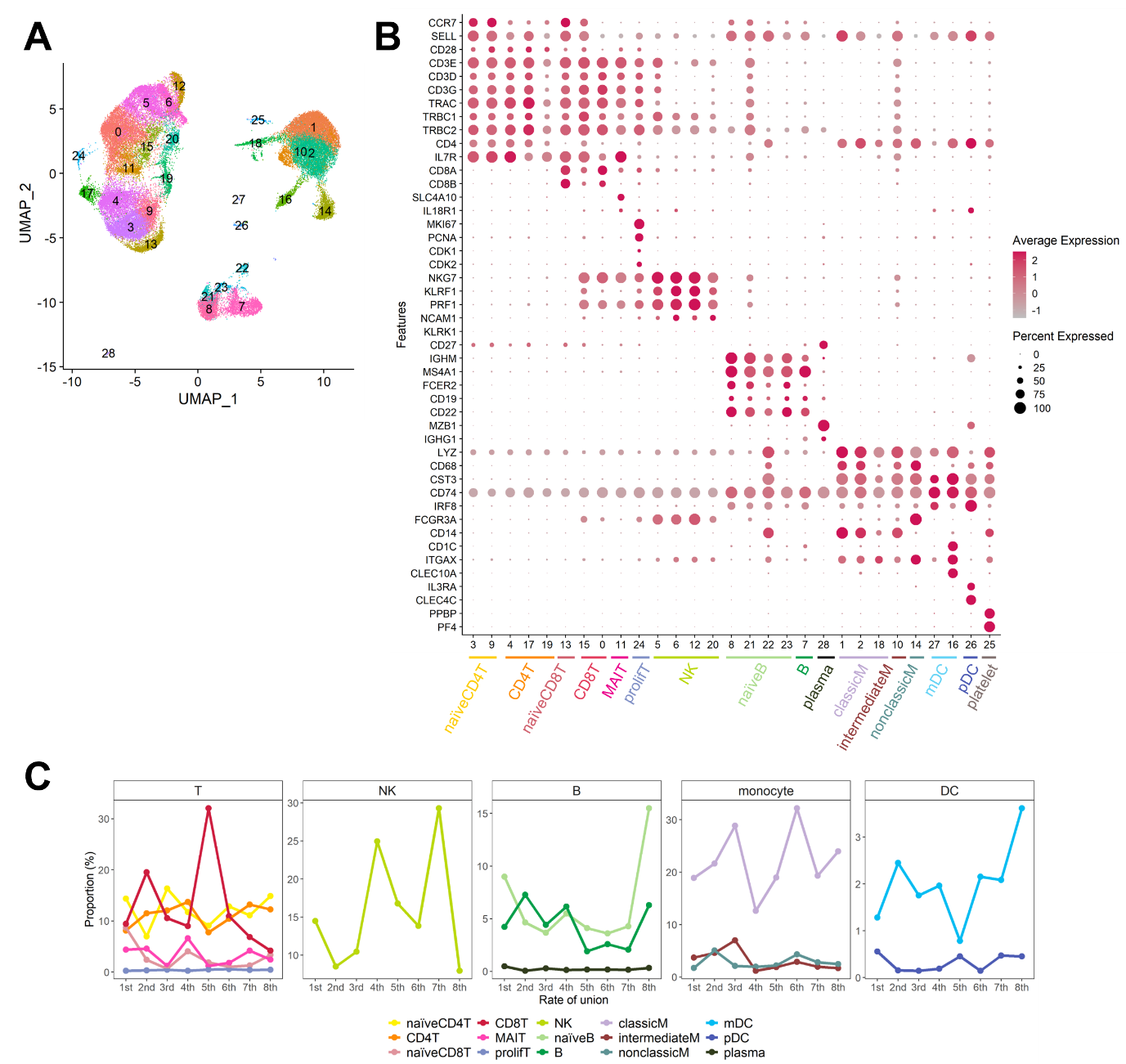
**

**Supplementary figure 1. Annotation of cell clusters obtained from preoperative patients.** (A) Dimensionality reduction visualization of clusters derived from PBMCs collected prior to surgical intervention. (B) Expression level of immune cell canonical markers in preoperative PBMC clusters. The colors of cell types correspond to those of annotated cell types, in Fig 2A. The markers for each cell identity are as follows: naive T (CCR7, SELL, CD28); T (CD3E, CD3D, CD3G, TRAC, TRBC1, TRBC2); CD4T (CD4, IL7R); CD8T (CD8A, CD8B); MAIT marker (SLC4A10, IL18R1); proliferating cell (MKI67, PCNA, CDK1, CDK2); NK (NKG7, KLRF1, PRF1, NCAM1); naive B (CD27, IGHM, IGHD); B (MS4A1, FCER2, CD19, CD22); plasma (MZB1, IGHG1); monocyte (FCGR3A, CD14, CCR5, CX3CR1, LYZ, CD68, CST3); mDC (CD1C, ITGAX, CLEC10A); pDC (IL3RA, CLEC4C); platelet (PPBP,PF4). (C) Fraction of cell identities before surgery according to the union rates. The patients on x axis are ordered by union rates from early union rate to late union rate. The cell identities are categorized by T, NK B, monocyte and DC lineages.

**
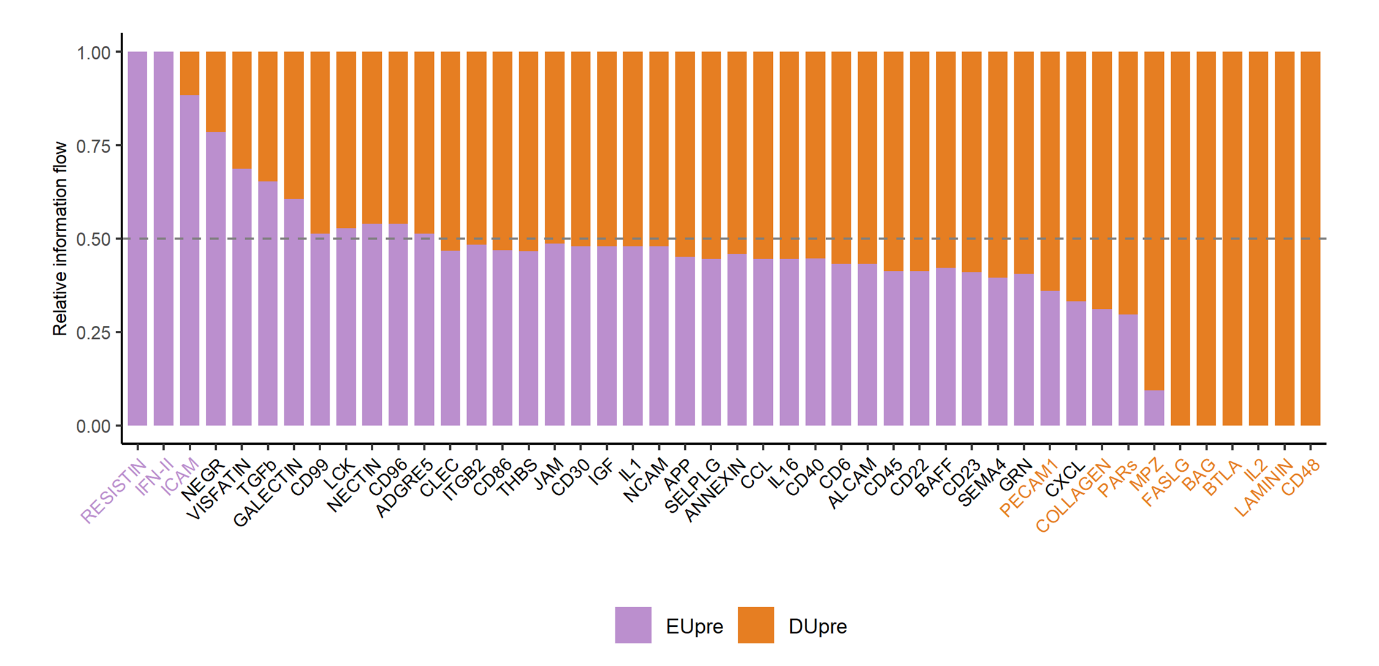
**

**Supplementary figure 2. Intensity of intercellular signaling in preoperative patients**

The color of the pathways reflects the enrichment of signals. The enhanced signals in the preoperative EU group are represented in purple, while those in the preoperative DU group are shown in orange. Signals that do not exhibit significantly different contributions are colored black.

**
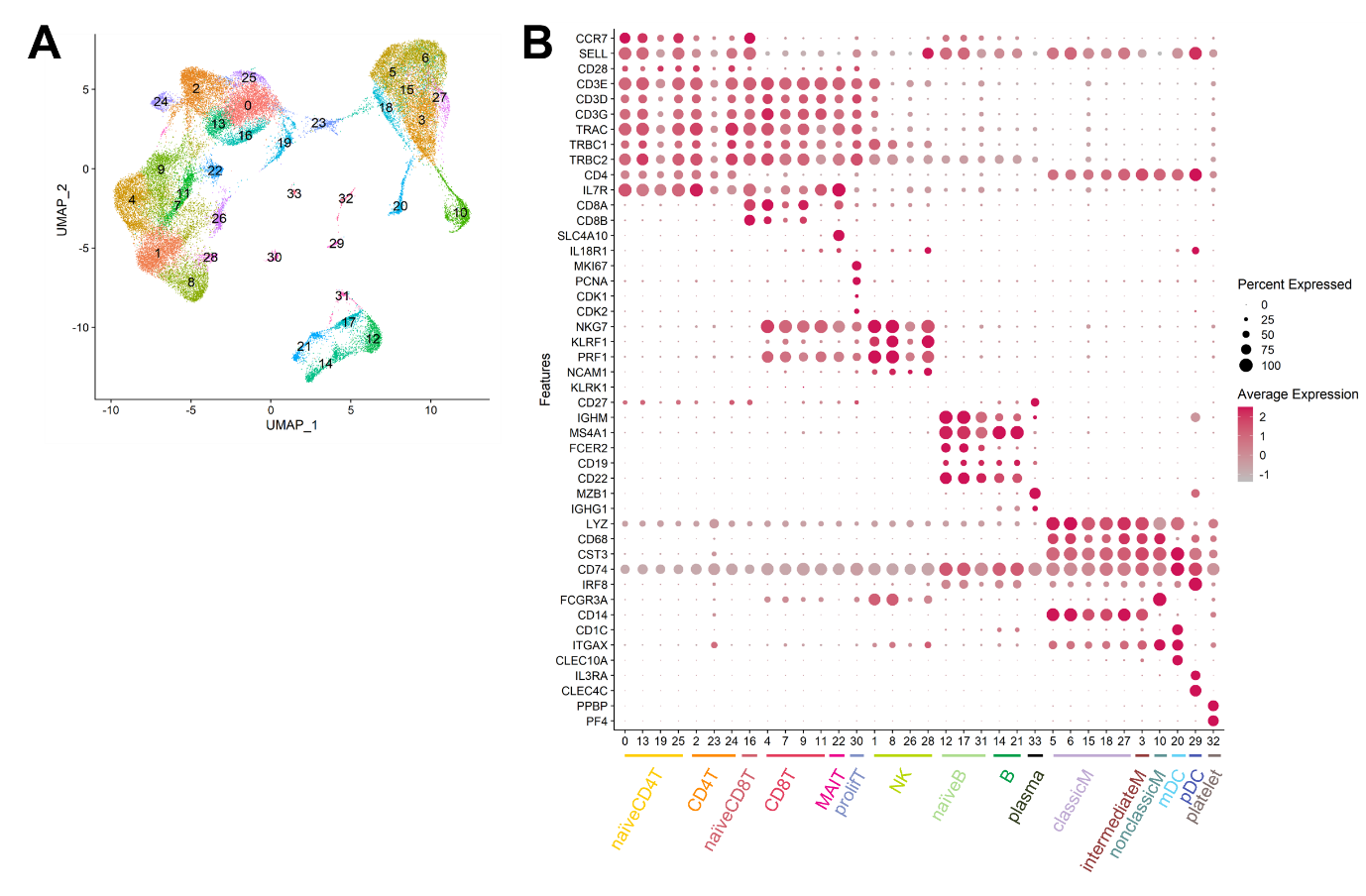
**

**Supplementary figure 3. Identification of cell identities obtained from postoperative patients.**

(A) Dimensionality reduction visualization of clusters derived from PBMCs collected after surgical intervention. (B) Expression level of immune cell canonical markers in postoperative PBMC clusters. The colors of cell types correspond to those of annotated cell types, in Fig 5A. The markers are identical to those detailed in Supplementary figure 1B.


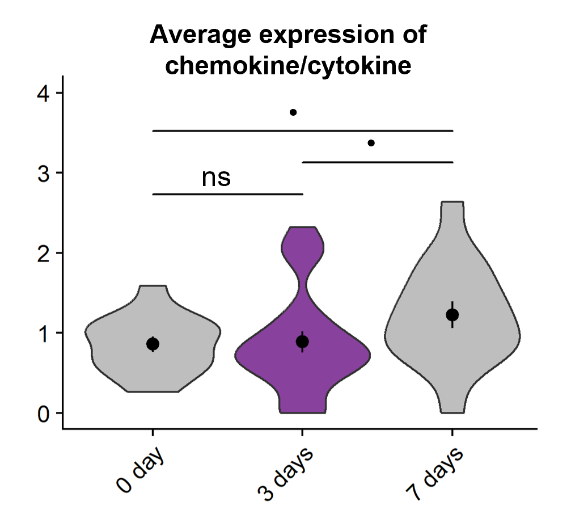


**Supplementary figure 4. Violin plot showing the average transcriptional levels of granzyme/granule within NK cells**. The p-values are as follows: • < 0.1, p>0.1: not significant (n.s.).
