# Supplementary material for: Deciphering the Osteoimmune Landscape in Subtalar Arthrodesis: A Single‐Cell RNA Sequencing Approach
Source: J Cell Mol Med. 2025 Dec 11;29(23):e70980. doi: 10.1111/jcmm.70980 (PMC12698338; doi:10.1111/jcmm.70980)
Supplement: Supplementary file 2 — Table S1: Clinical profiles of subjects. [file JCMM-29-e70980-s001.docx]

**Supplementary Table 1. Clinical profiles of subjects**

| **Patient No.** | **Age** | **Sex** | **Past**  **history** | **Medication** | **Immunosuppressant** | **Union rate** | **Union period** | **Category** |
| --- | --- | --- | --- | --- | --- | --- | --- | --- |
| 1 | 26 | Male | None | None | None | 100 | 8 weeks | Early union |
| 2 | 52 | Male | HT | Anti HT medication | None | 67 | 8 weeks | Early union |
| 3 | 72 | Male | HT, DM | Anti HT and DM medication | None | 69 | 10 weeks | Early union |
| 4 | 65 | Male | None | None | None | 61 | 10 weeks | Early union |
| 5 | 66 | Male | HT, DM, CHF | Anti HT, DM, and CHF medication | None | 50 | 10 weeks | Delayed union |
| 6 | 78 | Male | HT | Anti HT medication | None | 40 | 17 weeks | Delayed union |
| 7 | 59 | Male | None | None | None | 75 | 11 weeks | Early union |
| 8 | 50 | Male | DM | DM medication | None | 28 | 24 weeks | Delayed union |

CHF, Chronic Heart Failure; DM, Diabetes mellitus; HT, Hypertension
